# 

**Published:** 1909-12

**Authors:** 


Sixty-fifth Year.

VOL. LXV. DECEMBER, 1909. No. 5

### BUFFALO MEDICAL JOURNAL

Established 1845 by AUSTIN FLINT M. D.

EDITOR:

WILLIAM WARREN POTTER, M. D.

ASSISTANT EDITORS:

NELSON W. WILSON, M. D. JAMES WRIGHT PUTNAM, M. D.

ASSOCIATE EDITORS:

ERNEST WENDE, M. D. JOHN A. RAFTER, M. D.

JOHN PARMENTER, M. D. JOHN A. MILLER, Ph. D.

MAUD J. FRYE, M. D.

